# Statin-Related Necrotizing Autoimmune Myositis: More Than Myalgia

**DOI:** 10.7759/cureus.22654

**Published:** 2022-02-27

**Authors:** William J Scheuing, Frany B Dadhania, Adegbenga A Bankole

**Affiliations:** 1 Medicine, Carilion Clinic, Roanoke, USA; 2 Rheumatology, Virginia Tech Carilion School of Medicine, Roanoke, USA

**Keywords:** elevated liver-associated enzymes, dmards, intravenous immunoglobulins (ivig), subacute-onset muscle weakness, hmg-coa reductase inhibitors, statin-associated necrotizing autoimmune myositis, elevated ck levels, autoimmune neuromuscular disease, immune-mediated necrotizing myopathy, inflammatory myositis

## Abstract

Statins are widely prescribed for cardiovascular disease, and, in general, are well tolerated by most people. Most side effects related to statins are mild, with some side effects also considered a nocebo effect. Occasionally, statins can be associated with severe side effects. One of the more severe adverse events is immune-mediated necrotizing myositis, which is both difficult to diagnose and treat. The symptoms can be debilitating, and aggressive immunosuppressive therapy is the best-recognized method of treatment of this complication. In this case report, we discuss the clinical features, diagnosis, and treatment of this disease entity with an emphasis on the need for rapid diagnosis and aggressive treatment to help reduce morbidity.

## Introduction

Medications for cardiovascular disease are among the most common medications prescribed in the United States, and within this group, statins are among the most commonly prescribed medications [1]. Statins significantly reduce the risk of cardiovascular disease and are generally considered safe [2]. A significant portion of the side effects reported by patients can be related to the nocebo effect [3]; however, on rare occasions, statins can cause muscle disease, and most of these cases recover on discontinuation of the statin. Even more infrequently, statins can cause statin-associated necrotizing autoimmune myositis (SANAM) which is characterized by muscle necrosis on biopsy in the presence of antibodies to 3-hydroxy-3-methylglutaryl coenzyme A (HMG-CoA) reductase. Although these patients need treatment with aggressive immunosuppressive therapy, the treatment response is often poor with a variable clinical response. With the development of newer non-statin therapies for dyslipidemia, the prevalence of SANAM as a disease entity will decrease, making it even harder to diagnose and treat. Here, we present a typical case of SANAM with a poor response to aggressive therapy.

## Case presentation

Case history

A 72-year-old man from a skilled nursing facility (SNF) presented to the Emergency Department at Carilion Roanoke Memorial Hospital with a six-week history of progressive proximal symmetric muscle weakness, dyspnea on exertion, and a new lower extremity skin rash. He noted some difficulty rising from a seated position, climbing stairs, and lifting up his arms to 90 degrees independently. These limitations affected his ability to perform some activities of daily living including grooming and walking. He had no difficulty chewing, talking, swallowing, or opening and closing his eyes. He had diffuse muscle pain including in his proximal muscle groups in both limb girdles. In addition, he had joint pain, but he noted no swelling, redness, or warmth in his joints. He had no rash on his face, chest, back, hands, or on his eyelids, but did have a new lower extremity rash diagnosed as Grover’s disease after a biopsy performed by the dermatology consultation service. One week prior to this presentation, he was diagnosed with left lung basal pneumonia which was treated with oral antibiotics. He had fatigue, malaise, night sweats, and dyspnea on exertion. He did not have abdominal pain, change in bowel habits, or black or bloody stools. He did not have dysuria, difficulty voiding, or hematuria. At the time of admission, he was taking metoprolol succinate 50 mg daily, furosemide 20 mg daily, and aspirin 81 mg daily. He had been taking atorvastatin and sacubitril-valsartan for several years, but these medications had been discontinued at the onset of his muscle weakness. The statin had been started following the development of cardiac disease several years ago. As a result of his memory impairment, his spouse provided some needed details regarding his history.

He had a medical history of paraesophageal hiatal hernia, Grover’s disease, dyslipidemia, hypertension, coronary artery disease, heart failure, atrial fibrillation, and memory impairment. He lived in the SNF because of his memory impairment. His mother had been diagnosed with dermatomyositis at the age of 72.

His initial vital signs were normal. He had muscle atrophy in the shoulder and hip muscles, but no atrophy was noted in finger flexors. No muscle tremors or fasciculations were observed. His muscle strength was 3/5 in the right upper extremity and 2/5 in the left upper extremity. The power in his left and right hip flexors was 2/5. He had 5/5 power in his hands and fingers. His deep tendon reflexes were normal. The nail and nail fold capillaroscopy examinations were normal. His joint, pulmonary, and abdominal examinations were normal.

**Figure 1:**
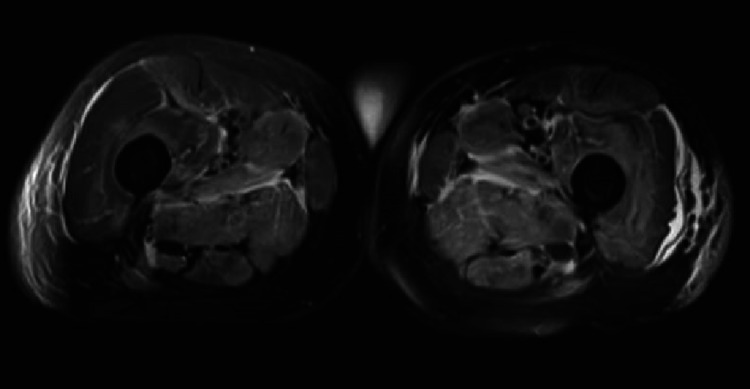
MRI of the bilateral lower extremities (quadriceps muscles) with and without intravenous contrast. There is enhancing feathery T2 hyperintense signal in the musculature of the proximal thighs bilaterally. Involvement is most pronounced in the adductor, obturator, and tensor fascia lata musculature with patchy involvement of the visualized proximal hamstrings and quadriceps musculature.

**Figure 2:**
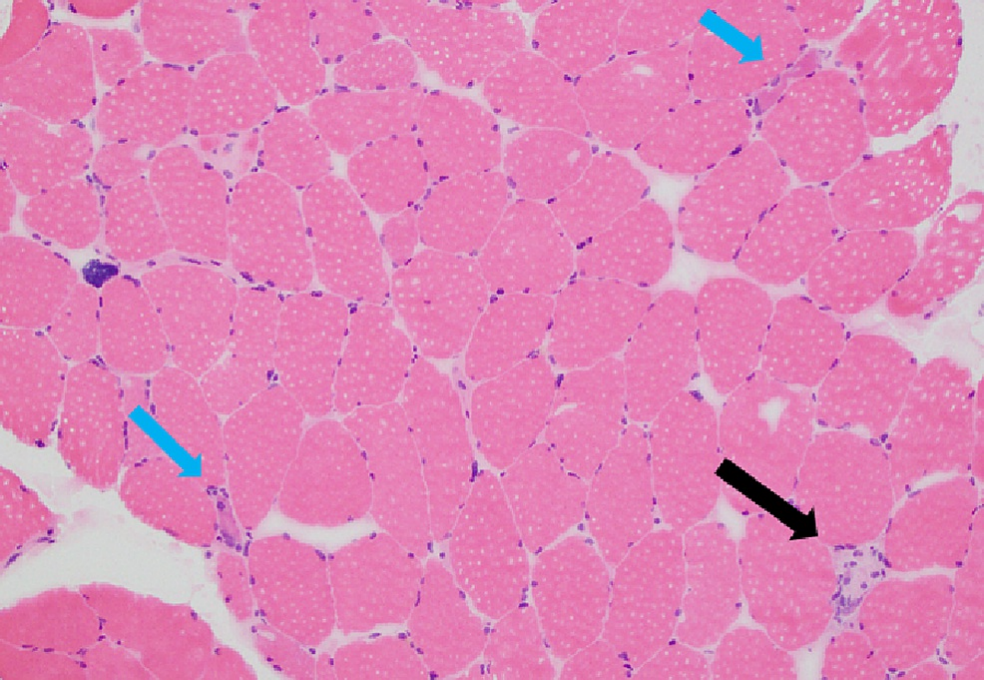
Muscle biopsy/Histology slide: hematoxylin and eosin stain (magnification power ×100). There was histologic evidence of necrotic foci with no significant inflammatory infiltrates or fibrosis. Note single necrotic fibers (black arrow) and regenerating fibers (blue arrow).

**Figure 3:**
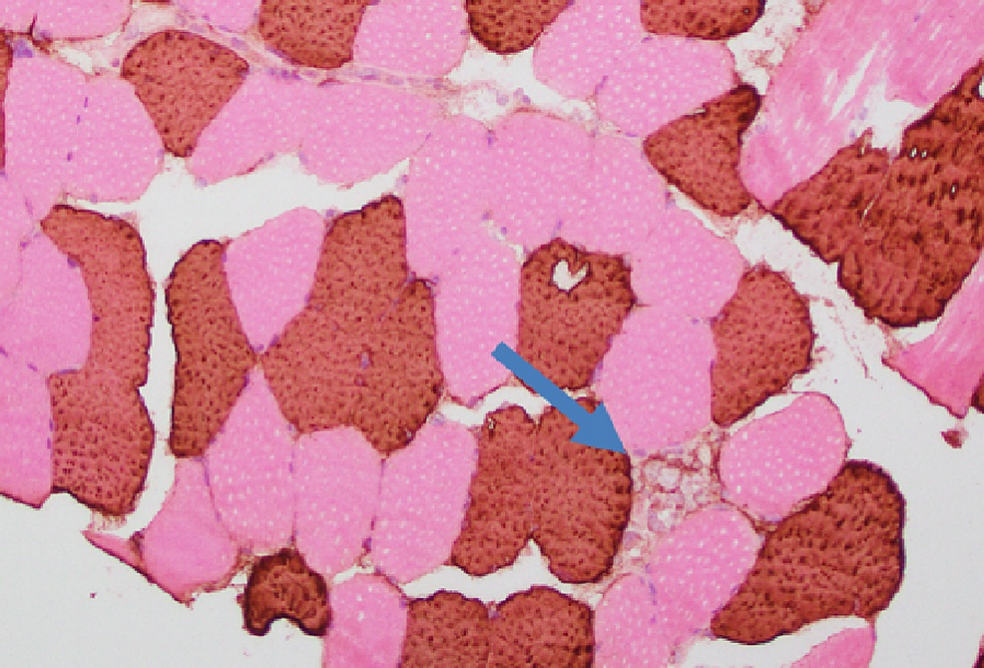
Muscle biopsy/Histology slide: immunostain for type 2 (fast) myosin (magnification power ×100). Type 2 fibers stain brown and type 1 fibers stain pink (eosin) counterstain. Multiple atrophic type 2 fibers are demonstrated, with a single necrotic fiber indicated by the arrow.

**Figure 4:**
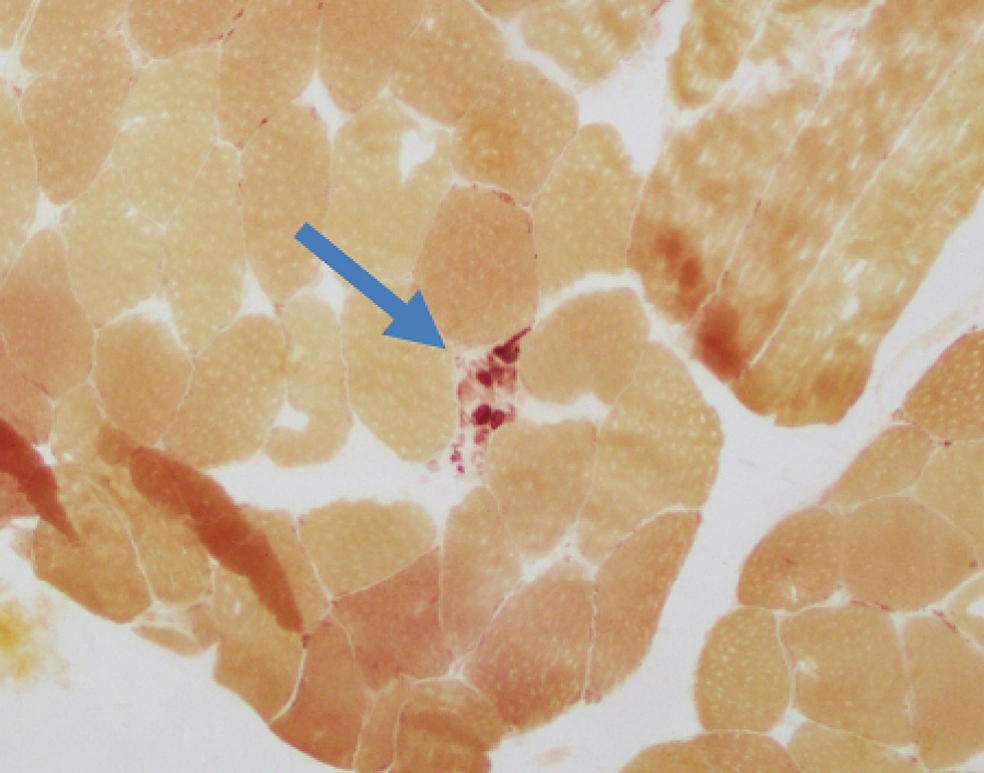
Muscle biopsy/Histology slide: esterase histochemical stain for esterase activity (magnification power ×100). Positive for esterase positive macrophage are within a necrotic myofiber, consistent with a necrotizing process.

The results of his laboratory tests are presented in Table 1. His blood tests confirmed an elevated creatine kinase (CK) level, as well as elevations in other muscle enzymes, including aspartate transaminase and alanine transaminase. He was treated with fluid hydration initially and had blood tests performed. A rheumatology consultation was requested as his CK did not respond to fluid therapy. A bilateral quadriceps muscle magnetic resonance imaging (MRI) study was performed (Figure 1), and an MRI-directed muscle biopsy was requested and performed (Figures 2-4). Based on the muscle enzyme levels, the strongly positive 3-hydroxy-3-methylglutaryl-coenzyme A reductase antibody (anti-HMGCR Ab), detected by enzyme immunoassay conducted at Quest Diagnostics reference labs, and the results of the muscle biopsy, he was diagnosed with SANAM.

**Table 1:** Admission and follow-up laboratory tests. Ab: antibody; EIA: enzyme immunoassay; H: high; CH: critical high

		1/2/2020	1/3/2020	1/4/2020	1/6/2020	5/11/2020
Component	Reference range					
Antisynthetase autoantibody: JO-1 Ab	<11 negative (EIA)			<11 SI		
Antisynthetase autoantibody: PL-7 Ab	<11 negative (EIA)			<11 SI		
Antisynthetase autoantibody: PL-12 Ab	<11 negative (EIA)			<11 SI		
Antisynthetase autoantibody: Ej Ab	<11 negative (EIA)			<11 SI		
Antisynthetase autoantibody: OJ Ab	<11 negative (EIA)			<11 SI		
Anti-signal recognition particle autoantibody (SRP Ab)	<11 negative (EIA)			<11 SI		
Mi-2 Alpha Ab	<11 negative (EIA)			<11 SI		
Mi-2 Beta Ab	<11 negative (EIA)			<11 SI		
Anti-melanoma differentiation-associated gene 5 antibody (MDA5 Ab)	<11 negative (EIA)			<11 SI		
Anti-human transcriptional intermediary factor antibody (TIF1-γ Ab)	<11 negative (EIA)			<11 SI		
nuclear matrix protein 2 autoantibody (NXP-2 ab)	<11 negative (EIA)			<11 SI		
Myeloperoxidase Abs	> or =1.0 Ab detected			<1		
Proteinase-3 Abs	> or =1.0 Ab detected			<1		
3-hydroxy-3-methylglutaryl-coenzyme A reductase antibody (Anti-HMGCR Ab)	<20 negative (EIA)			>200 (H)		
Creatine kinase	26–308 IU/L	17,559 (CH)		14,100 (CH)	20,724 (CH)	703 (H)
Erythrocyte sedimentation rate	0–20 mm/hour	16				
C-reactive protein	<1.0 mg/dL	1.04 (H)				
Lactate dehydrogenase	135–214 IU/L	1383 (H)				
Aldolase	≤8.1 U/L		108.0 (H)			
Alkaline phosphatase (ALP)	42–150 IU/L	77	65	61	68	55
Aspartate transaminase (AST)	10–42 IU/L	512 (H)	462 (H)	407 (H)	401 (H)	30
Alanine transaminase (ALT)	10–60 IU/L	545 (H)	477 (H)	431 (H)	442 (H)	48

He was started on prednisone 60 mg per day (approximately 1 mg/kg), methotrexate (MTX) 20 mg weekly, along with folic acid 1 mg daily, with only a modest improvement in his muscle enzymes and weakness initially. His muscle weakness worsened as the prednisone was rapidly tapered over six weeks following his transfer to an inpatient rehabilitation facility, with plans for adding rituximab. He followed up in the rheumatology clinic, and his prednisone was reinstituted and tapered over 16 weeks with a sustained improvement in his CK levels. Although rituximab was considered, it was never given as his wife opted for comfort care only, though she was happy to continue the prednisone given his clinical improvement. Though intravenous immunoglobulin (IVIG) was considered, it was not used in this case given the number of treatments monthly that would be needed, placing quite a burden on this patient with memory problems who resided in an SNF.

His wife opted for comfort measures at his last visit (which was a video visit as a result of the coronavirus disease 2019 pandemic) as he had memory impairment, poor mobility, and reduced oral intake prior to the development of SANAM and her concerns about the novel coronavirus. She wanted to continue MTX but did not want to add anything more.

## Discussion

Statins are very commonly prescribed for dyslipidemia and coronary artery disease and are generally considered safe [2]. They have anti-inflammatory properties and other properties that are beneficial in the treatment of a wide range of cardiovascular diseases [3]. Though the side effect profile of statins is very good, with only mild side effects in most cases [2], musculoskeletal side effects are among the more commonly reported side effects; however, recent studies have demonstrated that a significant proportion of these complications are related to the nocebo effect [4].

Statin use can be associated with various muscle-related side effects, ranging from myalgia with normal CK levels to muscle necrosis. Elevations in CK should trigger an investigation for more severe complications such as SANAM [5]. Some patients with statin-induced CK elevation improve with discontinuation of the statin [6], but those with SANAM only improve with immunosuppressive therapy. Even with treatment, the outcomes can be poor [7]. Although the pathogenesis of SANAM is not fully understood, the presence of anti-HMG-CoA reductase autoantibodies is thought to be both specific and pathogenic [8]. There is a genetic risk with class II human leukocyte antigen allele DRB1*11:01 being strongly associated with the expression of HMG-CoA reductase expression and development of anti-HMG-CoA reductase autoantibodies even in patients not exposed to statins [7]. The levels of this autoantibody also correlate with the level of muscle weakness and CK elevation and can explain why with an anti-HMGCR Ab level of >200 our patient had such high CK levels. The rare pathological finding of upregulation of major histocompatibility complex-1 (MHC-1) expression on the sarcolemma surface of non-necrotic muscle fibers scattered diffusely throughout the endomysium led to the investigation of SANAM as an immune-mediated reaction to statins. MHC-1 is not normally expressed on the surface of the sarcolemma and is considered a marker of immune activation [9]. The expression of MHC-1 products in target cells is a prerequisite for the cytolytic action of cytotoxic T cells, which destroy muscle fibers via the perforin pathway. Statins can also cause a self-limiting necrotic toxic effect; however, immune findings such as MHC class I upregulation in mature muscle fibers and anti-HMGCR Ab are absent.

With SANAM, prompt and immediate discontinuation of the statin drug is required if the patient is being treated with it. Following discontinuation of the statin, aggressive immunosuppressive treatment is needed, though a clinical improvement is not always noted [7]. There have been no randomized clinical trials, Food and Drug Administration-approved therapies, or widespread agreed-upon treatment protocols for SANAM. Moreover, clinical and therapeutic decisions are based on case reports, cohort studies, and clinical experience and expertise. Oral prednisone at a dose of 1 mg/kg of body weight per day is usually the initial therapy. Glucocorticoids have long been used in rheumatology for a rapid clinical response but are associated with numerous side effects [10], necessitating the use of steroid-sparing therapy such as MTX, azathioprine, or mycophenolate mofetil [6]. IVIG therapy is considered a first-line therapy [6,11] for SANAM as it can be used as part of a steroid-free treatment protocol [12], which may be important in patients with comorbidities such as diabetes. Rituximab can also lower muscle enzyme levels and allows for the discontinuation of other medications such as IVIG and steroids [13]. The presence of anti-HMGCR Ab following successful treatment indicated by return of normal strength and normalization of CK levels, as well as limited effect of plasmapheresis and rituximab, may indicate that the persistence of the autoantibody does not indicate ongoing disease [14].

Our case is a typical presentation of statin-associated necrotizing myopathy and highlights its poor and incomplete response to immunosuppressive therapy. SANAM must be considered in both male and female patients, in the right clinical scenario, and if patients do not respond as expected [14]. Given the uniqueness and specificity of the anti-HMGCR Ab [15], screening patients with this antibody test may prevent the need for more invasive testing. Over time, the continued development of monoclonal antibody therapies that lower cholesterol may reduce the incidence of SANAM. However, given the current ubiquitous use of statins, all physicians should be aware of SANAM, and the fact that the presence of anti-HMGCR Ab can be diagnostic and prevent the need for biopsy and other invasive procedures [14]. Furthermore, early diagnosis allows for early and aggressive treatment which improves the likely outcomes for these patients.

## Conclusions

Although statins are overall very safe, the frequency with which we use this class of drugs means that many of us will encounter patients with side effects. Although most of these side effects are mild, it is important that we are not complacent as on rare occasions we may come across patients with side effects that are medically significant causing considerable morbidity and mortality. If there are suspicions of musculoskeletal complications, including the possibility of immune-mediated myositis, a full history and physical examination must be performed to evaluate this. If the suspicion is supported by clinical findings, then an early referral to rheumatology should be made as aggressive immunosuppressive therapy may be required, as in our case.

This case also highlights the fact that the risks and benefits of all therapies, including commonly prescribed medications, must be considered. Even with the use of aggressive immunosuppressive therapy, as in our patient, the outcomes can be poor in cases of SANAM. More research is needed in this area of patient care.
